# Direct Numerical Simulation of Gas-Liquid Drag-Reducing Cavity Flow by the VOSET Method

**DOI:** 10.3390/polym11040596

**Published:** 2019-04-02

**Authors:** Yi Wang, Yan Wang, Zhe Cheng

**Affiliations:** 1 National Engineering Laboratory for Pipeline Safety/MOE Key Laboratory of Petroleum Engineering/Beijing Key Laboratory of Urban Oil and Gas Distribution Technology, China University of Petroleum, Beijing 102249, China; czheysin@163.com; 2 Institute of Naval Engineering Design, Naval Research Academy, Beijing 100070, China; wangyan6670390@163.com

**Keywords:** direct numerical simulation, drag reduction, gas-liquid flow, VOSET, turbulence

## Abstract

Drag reduction by polymer is an important energy-saving technology, which can reduce pumping pressure or promote the flow rate of the pipelines transporting fluid. It has been widely applied to single-phase pipelines, such as oil pipelining, district heating systems, and firefighting. However, the engineering application of the drag reduction technology in two-phase flow systems has not been reported. The reason is an unrevealed complex mechanism of two-phase drag reduction and lack of numerical tools for mechanism study. Therefore, we aim to propose governing equations and numerical methods of direct numerical simulation (DNS) for two-phase gas-liquid drag-reducing flow and try to explain the reason for the two-phase drag reduction. Efficient interface tracking method—coupled volume-of-fluid and level set (VOSET) and typical polymer constitutive model Giesekus are combined in the momentum equation of the two-phase turbulent flow. Interface smoothing for conformation tensor induced by polymer is used to ensure numerical stability of the DNS. Special features and corresponding explanations of the two-phase gas-liquid drag-reducing flow are found based on DNS results. High shear in a high Reynolds number flow depresses the efficiency of the gas-liquid drag reduction, while a high concentration of polymer promotes the efficiency. To guarantee efficient drag reduction, it is better to use a high concentration of polymer drag-reducing agents (DRAs) for high shear flow.

## 1. Introduction

Drag reduction by polymers was first discovered by Toms in 1948 [1]. It can largely reduce frictional forces in pipe flow by adding only a small amount of macro-molecular polymers, thus that the polymeric drag reduction can greatly save pumping energy (as high as 80%~90%) [2]. Due to the attractive energy-saving effect, drag reduction by polymer drag-reducing agents (DRAs) has received a lot of attention in the past several years in single-liquid turbulent flow systems. Burger et al. [3] reported the first commercial application for high polymer drag reduction in a Trans-Alaska oil pipeline. Virk obtained a quantitative relation between polymer concentration and drag reduction rate [4]. Warholic et al. [5] included the effect of Reynolds number and mixing. Housiadas and Beris [6] did the systematic DNS study for the elasticity and inertia of turbulent drag reduction using polymer. Li et al. [7] studied the effects of the Weissenberg number, polymer chain extensibility, and Reynolds number on drag reduction via the DNS of polymer induced drag reducing turbulent flow. Wang et al. [8] firstly introduced the wavelet method into the study of flow structures of polymer drag-reducing flow. Cai’s group studied polymer effects on turbulent Rayleigh-Benard convection and oscillating grid turbulence by both numerical simulation and experiment using particle image velocimetry [9,10,11].

Turbulent flow in the form of gas-liquid two phases is more complex than that in the form of a single phase [12], especially for oil and gas pipeline transportation in petroleum engineering. Therefore, the potential of drag reduction in two-phase turbulent flows can be larger than that in single-phase flows. However, studies on gas-liquid two-phase drag reduction are very limited compared with those on single-phase liquid drag reduction.

The first two-phase drag reduction study was made by Oliver and Yang [13] in 1968. They found the interfacial wave was suppressed by the macro-molecular-polymeric drag reducer. Greskovich and Shrier [14] reported the drag reduction rate 40%–50% in air-water slug flow. Sylvester and Brill [15] studied the air-water drag-reducing flow in a horizontal pipe using 100 ppm polyoxyethylene. They obtained a 35% drag reduction rate. Kang and Jepson [16,17] and Daas et al. [18] found the drag reduction rate of gas-liquid two-phase flow varied from 30% to 50% for the concentration of the high polymer increasing from 10 ppm to 50 ppm, respectively. For higher superficial gas velocity, the drag reduction rate drops from 50% to 30%. Kang et al. [19] also found the transition from the slug flow to stratified flow by adding 50 ppm drag reducing agents. Al-Sarkhi et al. [20,21,22] summarized from their experimental results that higher drag reduction usually occurs in the transition from slug or annular flow to stratified flow using the polymer drag-reducing agents. More recent studies can be found in references [23,24,25]. All these authors described many important factors on the drag reduction rate of gas-liquid turbulent flow. However, all the findings are based on observations of experimental phenomena, which can only give bulk features of two-phase drag-reducing flow. Representation of local characteristics has not been reported to the best knowledge of the authors. Lack of knowledge of local behavior for the two-phase drag-reducing flow induced by polymer additives restricted the reveal of the mechanism of two-phase drag reduction, although the mechanism of single-phase drag reduction has been discussed extensively. Thus, the mechanism of the two-phase drag reduction is still very unclear, which affects the application of the two-phase drag reduction.

To reveal the two-phase drag reduction mechanism, direct numerical simulation (DNS) is a potentially powerful tool. It has been developed in the field of turbulent flow and single-phase drag-reducing flow for several years but has not been seen in the field of two-phase drag-reducing flow. The main difference between DNS of single- and two- phase drag-reducing flow is the capture of the two-phase interface. The main methods of interface capture are volume-of-fluid (VOF) [26,27,28,29] and level set (LS) [30,31,32,33,34,35,36,37]. VOF can maintain the mass conservation of computation but the resolution of the complex interface is not accurate. LS can capture the interface precisely but cannot maintain the mass conservation. A coupled volume-of-fluid and level set method (VOSET) was proposed by Sun and Tao in 2010 [38]. This method can capture the two-phase interface accurately and maintain the mass conservation of computation thus that it receives very good commons and wide applications [39,40,41,42,43]. In two-phase drag-reducing flow, the interface capture is more difficult than the previous studies because the interface becomes highly irregular under turbulent fluctuation. Therefore, the VOSET method can be the best choice.

In this study, we aim to establish a DNS framework (governing equations and numerical methods) of gas-liquid drag-reducing flow based on the VOSET method and the research experience on DNS of single-phase drag-reducing flow. Then, we use this framework to obtain numerical results and try to explain the mechanism of two-phase drag reduction from the results.

## 2. Governing Equations

For incompressible immiscible gas-liquid drag-reducing flow, the straightforward way to establish the governing equations is considering the polymer effect in the momentum equation of the two-phase flow as follows:

(1) $$\frac{\partial \vec{u}}{\partial t}+\vec{u}\cdot \nabla \vec{u}=\frac{1}{\rho \left(\phi \right)}\left\{-\nabla p+\nabla \cdot \mu (\phi )\left[\left(\nabla \vec{u}\right)+{\left(\nabla \vec{u}\right)}^{T}\right]+\vec{F_{s}(\phi )}+\nabla \cdot \dot{\tau }\right\}$$

where $\vec{u}\left(=u\vec{i}+v\vec{j}\right)$ is the velocity vector with the component *u* in the *x* direction and *v* in the *y* direction in Cartesian coordinates, *p* is pressure, ρ and μ are density and dynamic viscosity of gas or liquid, respectively, which can be determined as:

(2) $$\rho \left(\phi \right)=H\left(\phi \right)\rho_{l}+\left(1-H\left(\phi \right)\right)\rho_{g}$$

(3) $$\mu \left(\phi \right)=H\left(\phi \right)\mu_{l}+\left(1-H\left(\phi \right)\right)\mu_{g}$$

where the subscripts “*l*” and “*g*” represent liquid and gas, respectively. $\rho_{l}$ is constant in the liquid phase while $\rho_{g}$ is constant in the gas phase, since the flow is incompressible. For a local area (e.g., one grid cell) containing both liquid and gas, ρ(ϕ) is used to calculate the density of the mixture thus that the fluid velocity can be easily calculated. The variation of ρ(ϕ) is only due to phase dynamics, not due to the variation of gas or liquid density. H(ϕ) is the smoothed Heaviside function:

(4) $$H(\phi )=\left\{ \begin{array}{ll} 0 & \phi <-\varepsilon \\ \frac{1}{2}[1+\frac{\phi }{\varepsilon }+\frac{1}{\pi }\sin(\frac{\pi \phi }{\varepsilon })] & |\phi |\leq \varepsilon \\ 1 & \phi >\varepsilon \end{array}\right.$$

where ε=1.5Δx, Δx is the grid spacing. $\vec{F_{s}\left(\phi \right)}$ in Equation (1) represents surface tension, which can be expressed as follows:

(5) $$\vec{F_{s}\left(\phi \right)}=-\sigma \kappa \left(\phi \right)\frac{dH\left(\phi \right)}{d\phi }\nabla \phi$$

where σ is the surface tension coefficient, κ(ϕ) is the curvature of the two-phase interface which can be calculated using Equation (6):

(6) $$\kappa \left(\phi \right)=\nabla \cdot \left(\frac{\nabla \phi }{\left|\nabla \phi \right|}\right)$$

All the terms in Equations (2)–(6) are related to the signed distance function ϕ, which is defined as:

(7) $$\phi =\left\{ \begin{array}{ll} -d & if\;\theta >0.5\\ 0 & if\;\theta =0.5\\ d & if\;\theta <0.5 \end{array}\right.$$

where *d* is the smallest distance from a grid cell to the two-phase interface, θ is the gas volume fraction. Its transport equation is:

(8) $$\frac{\partial \theta }{\partial t}+\nabla \cdot (\vec{u}\theta )=0$$

Polymer effect is represented by the last term in Equation (1), where $\dot{\tau }$ is the additional stress induced by the polymeric drag-reducing agent. This stress is determined by the Giesekus constitutive equation:

(9) $$\dot{\tau }+\lambda \left(\frac{D\dot{\tau }}{Dt}-\dot{\tau }\cdot \nabla \vec{u}-{\left(\nabla \vec{u}\right)}^{T}\cdot \dot{\tau }+\frac{\alpha }{\eta }\left(\dot{\tau }\cdot \dot{\tau }\right)\right)=\eta \left(\nabla \vec{u}+{\left(\nabla \vec{u}\right)}^{T}\right)$$

where λ is the relaxation time of polymer molecules, α is the mobility factor, η is the dynamic viscosity of the polymer. The additional stress is usually related to the conformation tensor as follows:

(10) $$\dot{\tau }=\frac{1}{\lambda }\left(\dot{c}-\eta \dot{I}\right)$$

where $\dot{c}$ is the conformation tensor of the drag-reducing polymer, $\dot{I}$ is the identity matrix. η can be calculated through the ratio β:

(11) β=η/(μ+η)

where μ is the dynamic viscosity of the solvent. Besides the equations above, the flow should definitely obey the continuity equation:

(12) $$\nabla \cdot \vec{u}=0$$

## 3. Numerical Methods

The governing equations of the gas-liquid drag-reducing flow contain two key variables: surface tension and conformation tensor. Thus, numerical methods should be adopted in these two groups. For numerical methods of the surface tension, one can refer to the classical VOSET method [38]. For numerical methods of the conformation tensor, one can refer to the typical treatment in single-phase drag-reducing flow [44,45,46,47,48]. The momentum equation is solved using the IDEAL algorithm [49] with the adaptive time step, which is a semi-implicit method to ensure numerical stability. The adaptive time step is fulfilled by setting the Courant number as $C={\left|\vec{u}\right|}_{\max}\Delta t/\Delta x=0.01$. The authors made the computational program by the above numerical methods using FORTRAN coding language. It is important to note that Equation (8) is not directly solved because the numerical instability is hard to control. Instead, it is solved by computing the fluxes on the cell interfaces. For details, please refer to Appendix A and reference [38].

**Figure 1:**
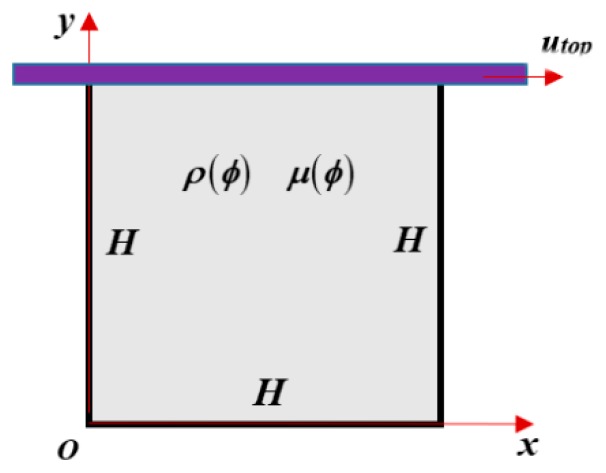
Lid-driven turbulent flow containing gas bubbles and drag-reducing polymers.

To test the governing equations and the numerical methods, a numerical case is designed for the DNS of the gas-liquid drag-reducing flow, as shown in Figure 1. Some literature showed that the lid-driven cavity case can be used for drag-reducing flow of nanofluid [50], two-phase flow with nanofluid [51,52], and mixed convection of a non-Newtonian fluid [53]. Thus, it is appropriate for two-phase drag-reducing flow induced by non-Newtonian polymer solution. The computational domain is a square cavity with the side length *H* and the moving wall velocity *u*_top_. *H* is set as 1 m with the mesh size 150 × 150. Other key parameters are set as: *λ* = 0.14 s, *β* = 0.8. The numerical test shows that the computation is restricted in a very narrow range (*u*_top_ = 1 m/s~2 m/s). When the *u*_top_ is larger than this range, the computation is broken up more quickly. This indicates that numerical stability is very weak in current numerical framework.

**Figure 2:**
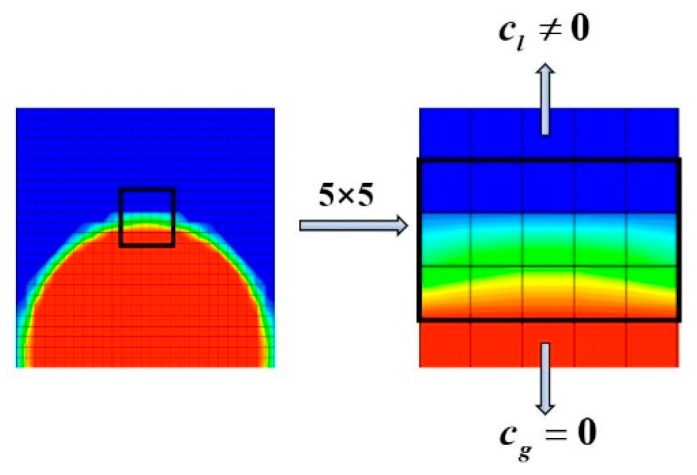
Conformation tensor near the two-phase interface.

For two-phase flow without polymer, the numerical stability is strong as proved in the previous reference. For single-phase polymeric drag-reducing flow, the numerical stability is also strong. Therefore, the reason for the instability of the two-phase drag-reducing flow may come from the interaction between the interface and the polymeric molecules. We analyzed the conformation tensor induced by the polymer near the gas-liquid interface (Figure 2) and realized that the conformation tensor in the gas phase is zero because the polymer only dissolves in the liquid phase. This causes a large jump of the conformation tensor near the interface, which can easily generate strong numerical instability. To let the jump be smoother, the conformation tensor is also defined as the function of the signed distance function ϕ:

(13) $$c\left(\phi \right)=H\left(\phi \right)c_{l}+\left(1-H\left(\phi \right)\right)c_{g}$$

From the definition of the Heaviside function (Equation (4)), it can be known that H(ϕ) is 0 when a grid cell is totally occupied by gas (ϕ<−ε) thus that $c\left(\phi \right)=c_{g}=0$. When a grid cell is totally occupied by liquid (ϕ>ε), H(ϕ) is 1 thus that $c\left(\phi \right)=c_{l}$. When a grid cell is occupied by both gas and liquid, $0<c\left(\phi \right)<c_{l}$. The analysis shows that the interpolation in Equation (13) ensures the physical meaning of the conformation tensor and makes it vary gradually near the two-phase interface instead of the jump. After this treatment, the DNS can be extended to the maximum *u*_top_ as large as 50 m/s, indicating that the numerical stability is greatly enhanced. Thus, the following DNS takes the improved numerical framework.

**Figure 3:**
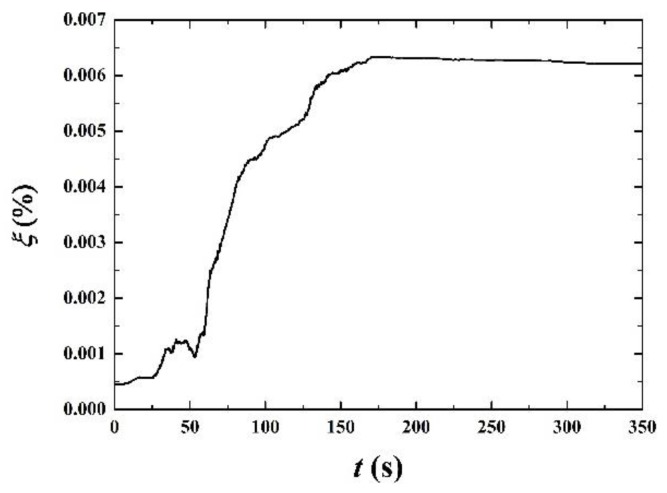
Examination of the phase conservation.

To examine the phase conservation, the numerical error between the theoretically whole gas volume fraction and computationally whole gas volume fraction at every time step is checked. The expression of the error is:

(14) $$\xi =\frac{\left|\sum_{j=1}^{ny}\sum_{i=1}^{nx}\theta_{i,j}-\sum_{j=1}^{ny}\sum_{i=1}^{nx}\theta_{i,j}^{T}\right|}{\sum_{j=1}^{ny}\sum_{i=1}^{nx}\theta_{i,j}^{T}}\times 100\%$$

where $\theta_{i,j}^{T}$ is the theoretical gas volume fraction. Its summation in the whole domain ($\sum_{j=1}^{ny}\sum_{i=1}^{nx}\theta_{i,j}^{T}$) should be constant for the incompressible flow. Thus, the summation can be calculated easily from the initial phase distribution. The error in different time is shown in Figure 3. It only has tiny variations between 0%~0.007%, verifying the phase conservation is very well satisfied in the computation.

## 4. Results and Discussion

The above numerical framework is used to do the DNS of the gas-liquid drag-reducing flow in the cavity shown in Figure 1. We wish to analyze the mechanism of drag reduction by polymer in gas-liquid two-phase flow. For the convenience of analyses, some characteristic variables are defined as follows:

(1) Bulk mean velocity:

(15) $$\bar{u}=\frac{\sum_{j=1}^{ny}\sum_{i=1}^{nx}\left(1-\theta_{i,j}\right)u_{i,j}}{\sum_{j=1}^{ny}\sum_{i=1}^{nx}\left(1-\theta_{i,j}\right)}$$

(16) $$\bar{v}=\frac{\sum_{j=1}^{ny}\sum_{i=1}^{nx}\left(1-\theta_{i,j}\right)v_{i,j}}{\sum_{j=1}^{ny}\sum_{i=1}^{nx}\left(1-\theta_{i,j}\right)}$$

(2) Reynolds number:

(17) $$Re=\frac{\rho_{l}H\bar{U}}{\mu_{l}}\;\bar{U}=\sqrt{{\bar{u}}^{2}+{\bar{v}}^{2}}$$

(3) Fluctuation intensity:

(18) $$u_{\mathrm{rms}}=\sqrt{\sum_{j=1}^{ny}\sum_{i=1}^{nx}{\left[\left(1-\theta_{i,j}\right)u_{i,j}-\bar{u}\right]}^{2}/\left(nx\cdot ny\right)}$$

(19) $$v_{\mathrm{rms}}=\sqrt{\sum_{j=1}^{ny}\sum_{i=1}^{nx}{\left[\left(1-\theta_{i,j}\right)v_{i,j}-\bar{v}\right]}^{2}/\left(nx\cdot ny\right)}$$

**Figure 4:**
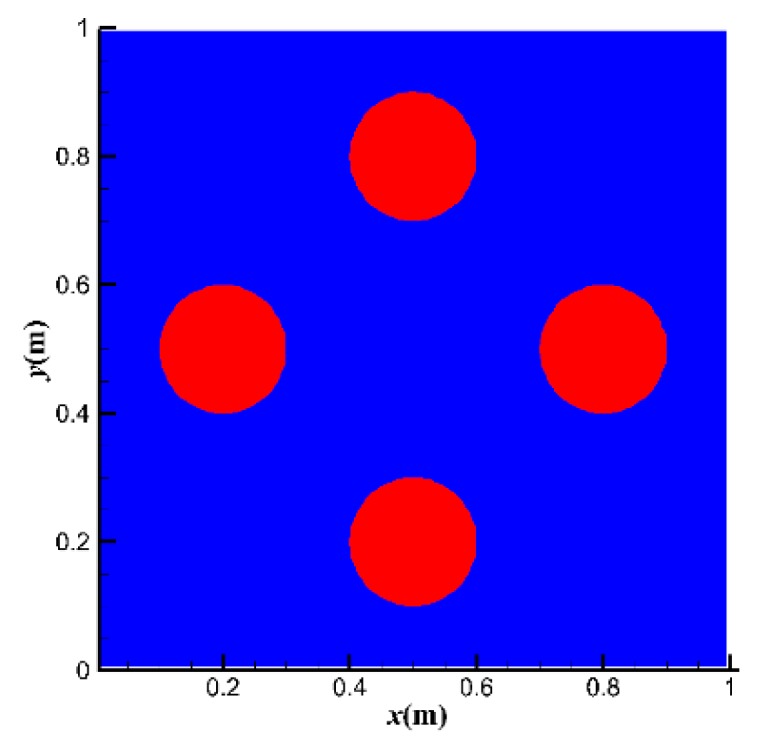
Initial distribution and size of the bubbles.

For lid-driven flow, it is difficult to define the drag reduction rate directly because it is hard to calculate the friction factor for this kind of rotational flow. Drag reduction can be represented not only by the reduction of the friction factor but also by the promotion of the bulk mean velocity. Thus, we used the increasing percentage of the bulk mean velocity with the addition of polymer under the same lid-driven velocity to measure the drag reduction quantitatively. Three lid-driven velocities (*u*_top_ = 1, 10, 50 m·s^−1^) were examined. The initial phase distribution is shown in Figure 4, where the initial bubble diameter is 0.2 m.

**Figure 5:**
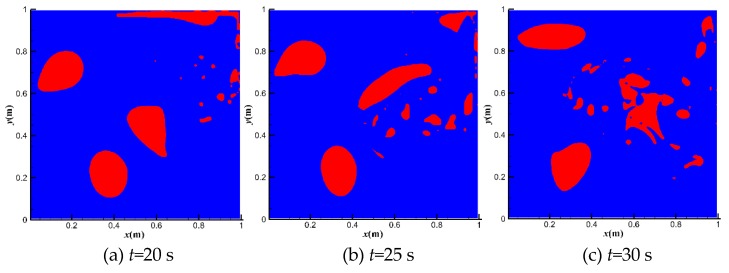

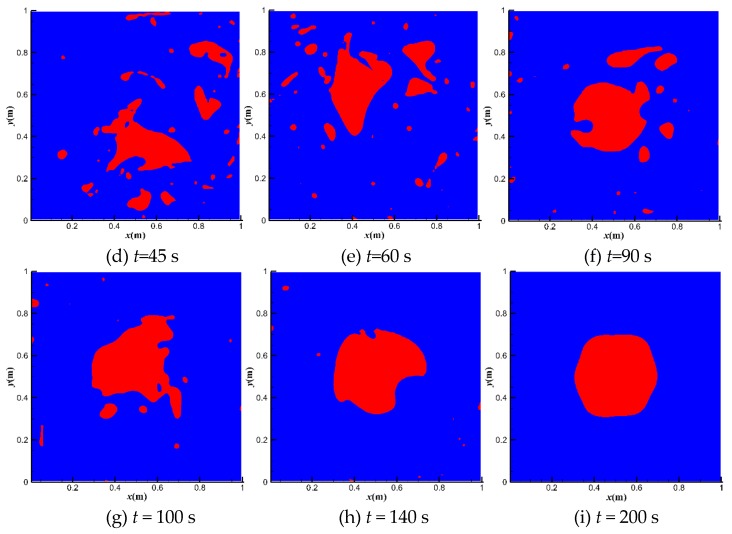
Bubble movements at *u*_top_ = 1 m·s^−1^.

Figure 5 shows the bubble distributions at different moments at *u*_top_ = 1 m·s^−1^. The red area is the gas phase, while the blue area is the liquid phase. The top right bubble breaks firstly due to its position in the high shear region. Other bubbles break quickly from 20 to 45 s. After 100 s, the scattered small bubbles merge into one large bubble in the center of the domain. The large bubble is quite stable due to its position in the low shear area.

**Figure 6:**
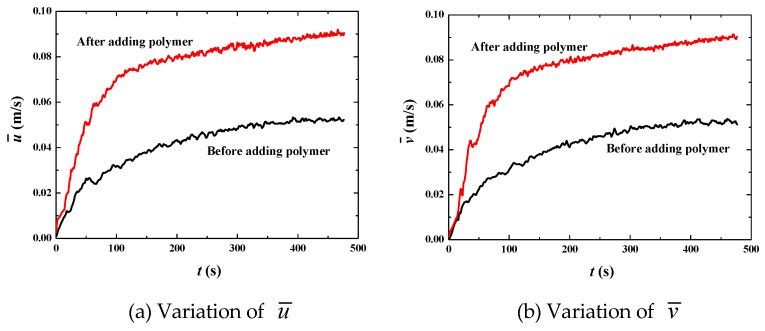

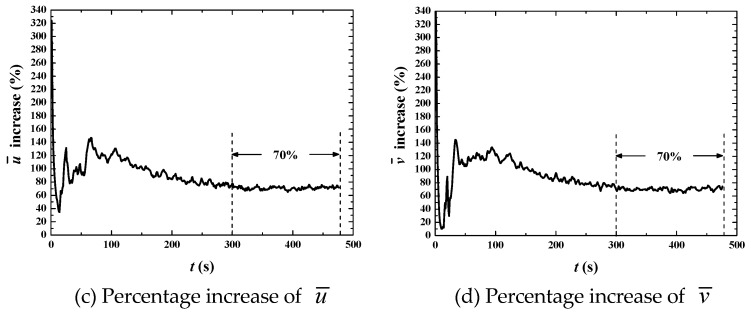
Increase of bulk mean velocity by adding polymer DRAs at *u*_top_ = 1 m/s.

**Figure 7:**
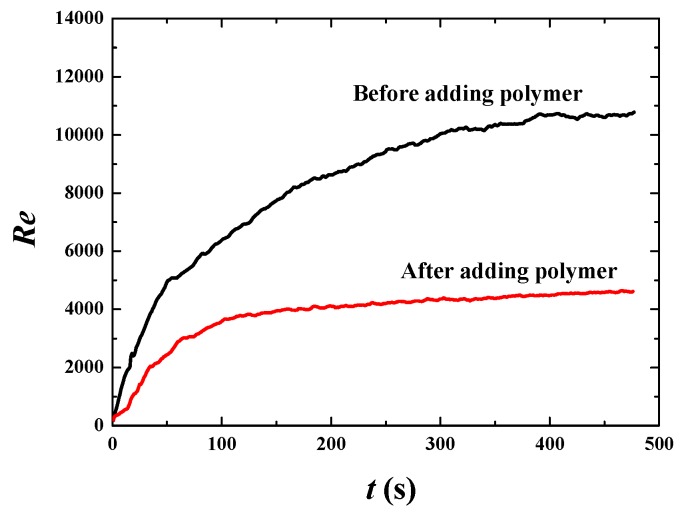
Reynolds numbers before and after adding polymer drag-reducing agents (DRAs) at *u*_top_ = 1 m·s^−1^.

Figure 6 shows that the bulk mean velocity ($\bar{u}$ in the *x* direction and $\bar{v}$ in the *y* direction) increases rapidly from the initial condition to about 300 s. After 300 s, the bulk mean velocity and its increasing percentage tend to stabilize, thus that fully developed flow is achieved in this time span (Figure 6a,b). Once we obtained the fully developed flow, some statistically stable parameters can be analyzed to reveal more essential mechanisms. The percentage increase of the bulk mean velocity for the fully developed flow is about 70% (Figure 6c,d), showing good drag reduction. Figure 7 shows that the Reynolds number decreases from about 10,000 to 4000, verifying that flows with or without polymer are both in the turbulent regime. The decrease of the Reynolds number in the situation of increasing mean velocity is due to the significant change of liquid viscosity. From Equation (11), it is easy to find that the dynamic viscosity of the polymer *η* is 4 times of the solvent viscosity *μ*, thus that the liquid viscosity *μ_l_* becomes 5 times of *μ* (*μ_l_* = *μ* + *η*). For the Newtonian flow without polymer, *μ_l_* = *μ*. That means liquid viscosity after adding polymer is 5 times than that without polymer. Thus, Reynolds number decreases dramatically according to Equation (17), although the bulk mean velocity increases by 70% due to adding polymer. The decrease in *Re* indicates the change of turbulent fluctuation intensity. Therefore, the fluctuation intensity should be analyzed to further demonstrate drag reduction behavior.

**Figure 8:**
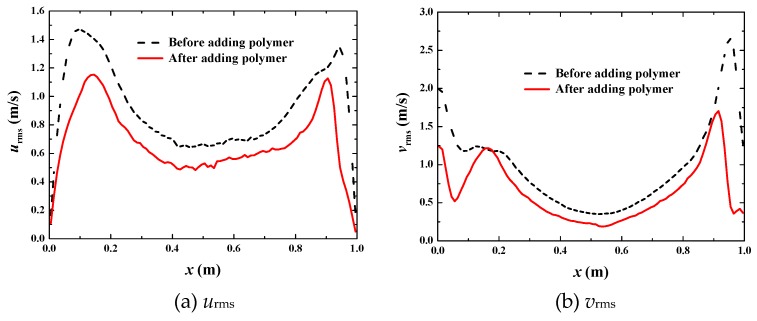
Comparison of fluctuation intensity at *u*_top_ = 1 m·s^−1^.

Figure 8 shows the fluctuation intensity. It is clear that the fluctuation intensity decreases at all points after adding polymer DRAs. The average percentage decrease is 26% for *u*_rms_ and 32% for *v*_rms_, respectively. The suppression mechanism of the fluctuation is the same as that in the single-phase drag-reducing flow.

**Figure 9:**
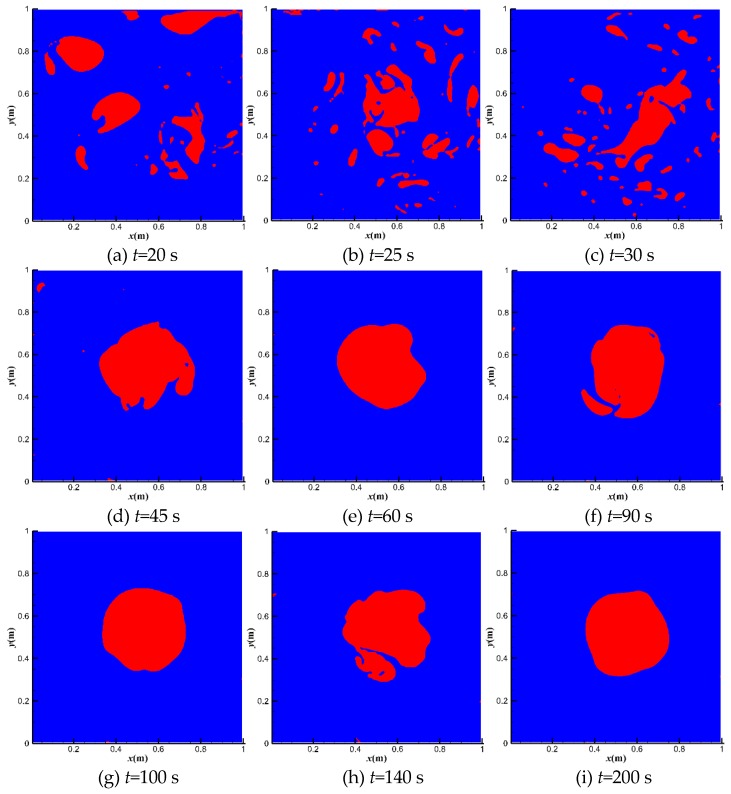
Bubble movements at *u*_top_ = 10 m·s^−1^.

**Figure 10:**
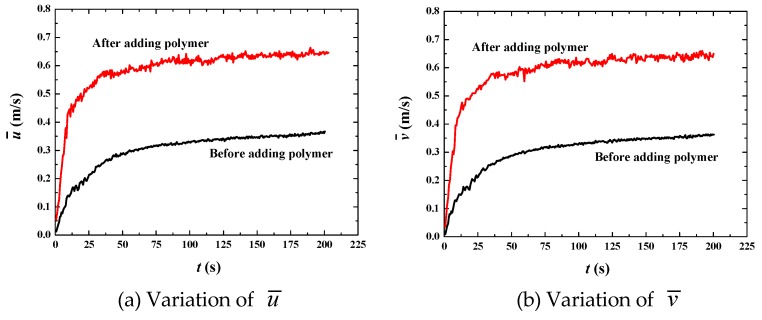

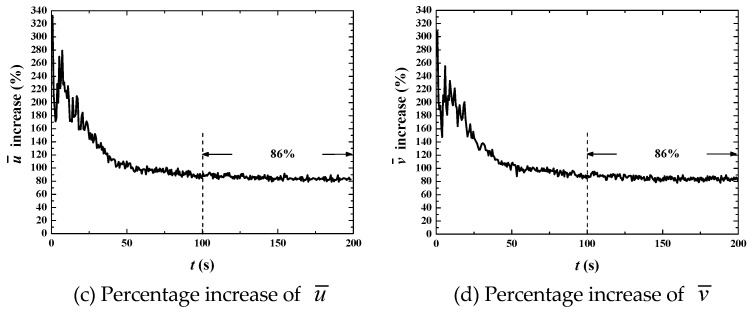
Increase of bulk mean velocity by adding polymer DRAs at *u*_top_ = 10 m·s^−1^.

**Figure 11:**
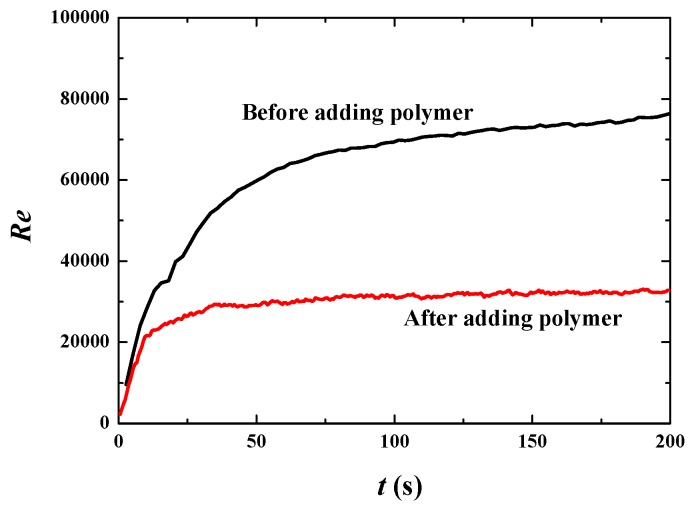
Reynolds numbers before and after adding polymer DRAs at *u*_top_ = 10 m·s^−1^.

Phase distribution along with time at *u*_top_ = 10 m·s^−1^ is shown in Figure 9. The break and merge of bubbles take a much shorter time (using only 45 s). This indicates larger inertia of the flow field for larger driven force. When the flow reaches full development, the bulk mean velocity increases by 86% by adding polymer DRAs (Figure 10). *Re* is about 7 × 10^4^ before adding polymer and about 3 × 10^4^ after adding polymer (Figure 11), which is much larger than the case of *u*_top_ = 1 m·s^−1^. This indicates a higher drag reduction for stronger turbulence.

**Figure 12:**
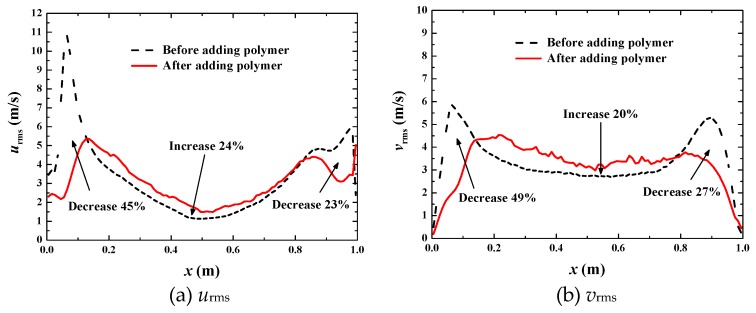
Comparison of fluctuation intensity at *u*_top_ = 10 m·s^−1^.

Fluctuation intensities are analyzed in Figure 12. It is interesting to find that the fluctuation intensities *u*_rms_ and *v*_rms_ are not all suppressed everywhere as in the case of *u*_top_ = 1 m·s^−1^. They are only suppressed in near wall regions (about 0~0.2 and 0.8~1) but enhanced in the core region (about 0.2~0.8). This phenomenon is quite different from single-phase drag-reducing flow, where the fluctuation intensities are all suppressed in the flow region. The enhancement of the fluctuation intensities in the core region is due to the periodic break and merge of the big bubble in the center of the cavity for the fully developed turbulent flow, as shown in Figure 9e–i. The enhancement in the core region causes on average a 24% increase of *u*_rms_ (Figure 12a) and an average 20% increase of *v*_rms_ (Figure 12b). The suppression in the near wall regions causes an average of 45% and 23% decrease of *u*_rms_ (Figure 12a) and an average of 49% and 27% decrease of *v*_rms_ (Figure 12b). The decrease is much larger than the increase, thus that the net effect is a decrease of the fluctuation intensities. This is the reason that drag reduction can still occur when the turbulent fluctuation is enhanced in the core region of the flow.

**Figure 13:**
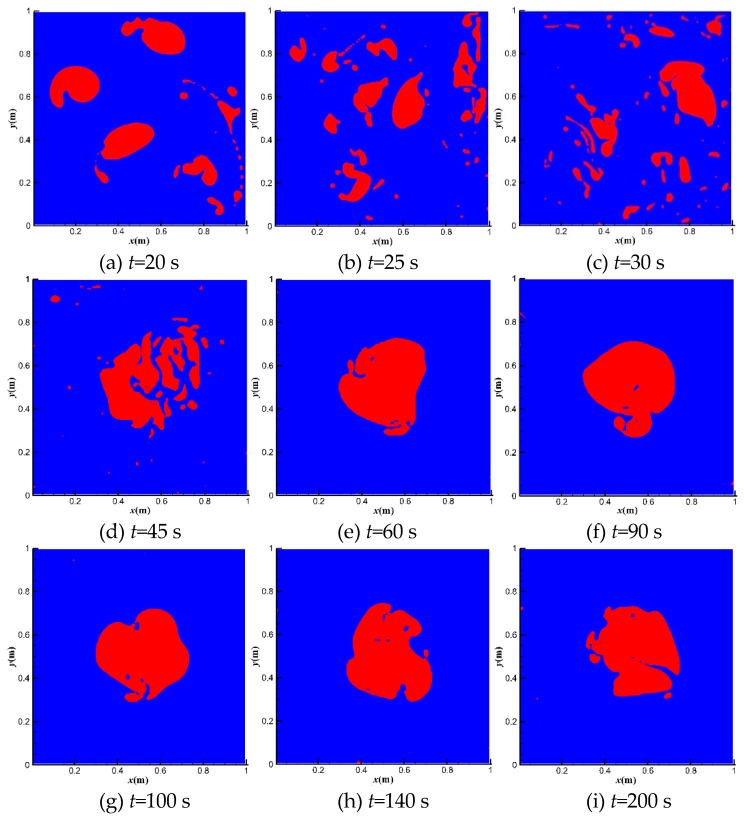
Bubble movements at *u*_top_ = 50 m·s^−1^.

**Figure 14:**
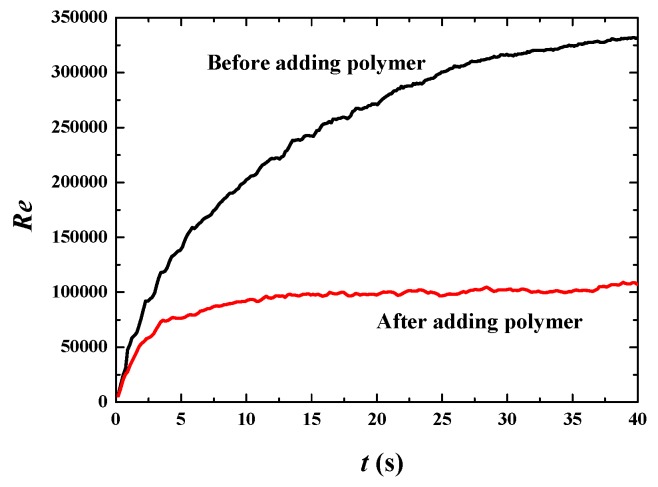
Reynolds numbers before and after adding polymer DRAs at *u*_top_ = 50 m·s^−1^.

**Figure 15:**
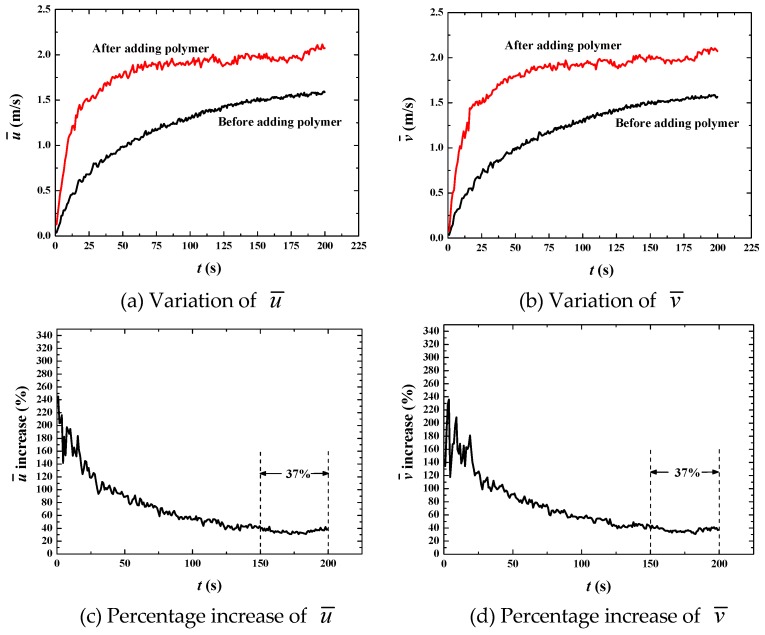
Increase of bulk mean velocity by adding polymer DRAs at *u*_top_ = 50 m·s^−1^.

**Figure 16:**
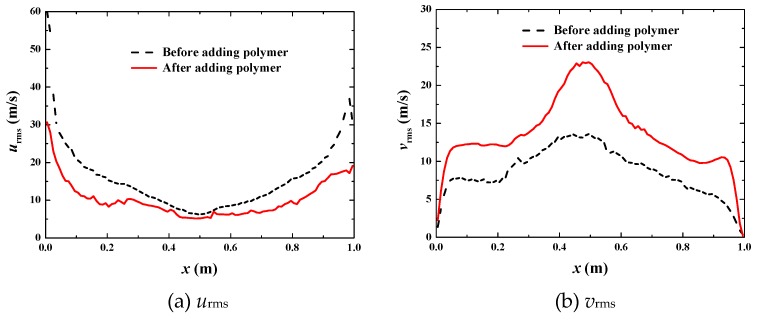
Comparison of fluctuation intensities at *u*_top_ = 50 m·s^−1^.

When *u*_top_ reaches up to 50 m·s^−1^, the bubbles are always in dynamic motions with frequent breaking and merging (Figure 13) because turbulence is very active with the Reynolds number around 3 × 10^5^ before adding polymer, and still as high as about 1 × 10^5^ after adding polymer (Figure 14). The bulk mean velocities also increases after adding polymer (Figure 15a,b), indicating drag reduction is still valid at a high Reynolds number. However, the drag reduction efficiency is much lower than the previous two cases. The increase in the bulk mean velocity is only 37% (Figure 15c,d). With further analysis of the fluctuation intensities, we find a phenomenon that *u*_rms_ is decreased by about 32% but *v*_rms_ is increased by about 62% (Figure 16). That means some of the fluctuations are not suppressed but enhanced. This is the reason for only a 37% increase in the bulk mean velocity.

The drop of the percentage from 86% to 37% indicates that the polymeric DRAs cannot sufficiently suppress all the turbulent fluctuations when the shear in the flow field is extremely high (large *u*_top_ corresponds to high shear). The reason may be the concentration of polymer is not sufficiently high relative to the extremely high shear. To verify this explanation, we do more DNS cases under different *β*. From the definition of *β* in Equation (11), it can be known that a higher *β* means higher viscosity proportion of polymer in the solution. Higher viscosity proportion can only be caused by adding more polymer DRAs. Therefore, *β* reflects the concentration of polymer. The percentage increase of the bulk mean velocity under different *β* and *u*_top_ are listed in Table 1.

With increasing *β* at a constant *u*_top_, the percentage always increases. This demonstrates that high concentration is helpful for two-phase drag reduction. With increasing *u*_top_ at a constant *β*, the percentage basically increases first and then decreases. This means high shear depresses two-phase drag reduction, thus that low drag reduction occurs in almost all cases (dark area in Table 1). Promotion of polymer concentration to a very high level, e.g., *β* = 0.95 in Table 1, can keep the high efficiency of drag reduction even if the turbulent shear is very high.

## 5. Conclusions

Direct numerical simulation of gas-liquid two-phase drag-reducing flow is fulfilled for the first time by combining the efficient interface tracking method VOSET and the constitutive model of polymer DRAs. Governing equations are established reflecting characteristics of the two-phase and polymeric flow. Numerical stability is enhanced by the proposed method thus that the robustness of the computation is largely promoted. The mechanism of the gas-liquid drag reduction is discussed based on the DNS results. Detailed conclusions can be summarized as follows:

(1) DNS of the polymeric drag reduction for gas-liquid turbulent flow is practical. Conformation tensor induced by the polymer should be smoothed near the two-phase interface to greatly enhance the numerical stability thus that the DNS can be made at much wider scopes of parameters. This is the first helpful attempt for the DNS of two-phase gas-liquid drag-reducing flow.
(2) The drag reduction mechanism of gas-liquid drag-reducing flow can be the global suppression of turbulent fluctuations. This is the same as a single-phase drag-reducing flow. The mechanism can also be due to local enhancement in the core region with local suppression near the walls of turbulent fluctuations. This is the special feature of gas-liquid drag-reducing flow.
(3) High shear of flow depresses the efficiency of the gas-liquid drag reduction, while a high concentration of polymer promotes the efficiency. To guarantee efficient drag reduction, it is better to use a high concentration of polymer DRAs.

## Figures and Tables

**Table 1:** Combination effect of shear and concentration on drag reduction

*β*	*u*_top_ = 1 m·s^−1^	*u*_top_ = 10 m·s^−1^	*u*_top_ = 50 m·s^−1^
0.4	20%	8%	0%
0.6	40%	45%	30%
0.8	70%	86%	37%
0.95	80%	87%	89%

**Figure A1:**
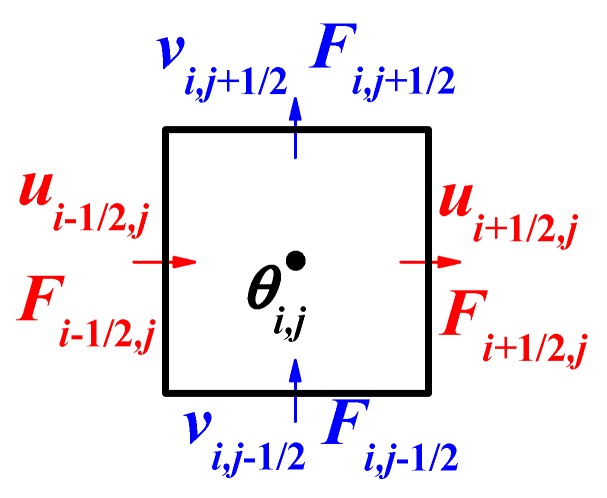
Illustration of variable dispositions of Equation (8).

**Figure A2:**
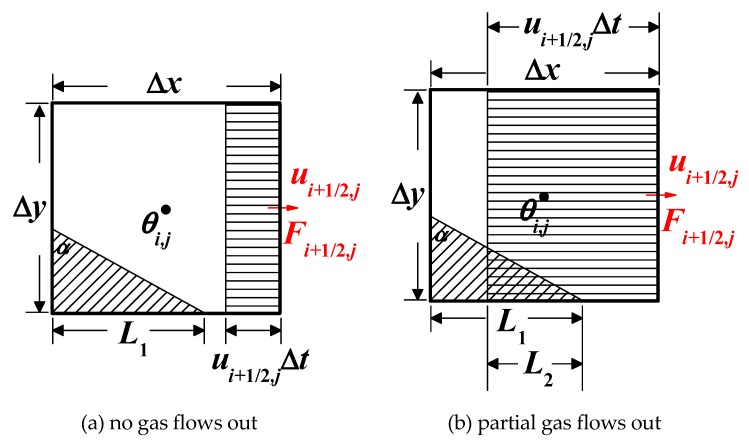
Determination of the gas flux with the two-phase interface in the cell.

## Appendix A

Gas volume fraction θ in Equation (8) is an important variable in the whole simulation. Direct computation of Equation (8) in an entirely traditional way may encounter strong numerical instability due to the lack of a diffusion term. The treatment is taking advantage of the two-phase interface. Details can be found in Sun’s paper [35]. Here, we explain the idea briefly in the form of our own case as follows.

Equation (8) can be discretized to be the following finite volume form:

(A1) $$\left(\theta_{i,j}^{\left(n+1\right)}-\theta_{i,j}^{\left(n\right)}\right)\Delta x\Delta y=F_{i-1/2,j}^{\left(n\right)}-F_{i+1/2,j}^{\left(n\right)}+F_{i,j-1/2}^{\left(n\right)}-F_{i,j+1/2}^{\left(n\right)}$$

where $F_{i-1/2,j}^{\left(n\right)},F_{i+1/2,j}^{\left(n\right)},F_{i,j-1/2}^{\left(n\right)},F_{i,j+1/2}^{\left(n\right)}$ are gas fluxes on the borders of the cell (*i*,*j*), which are illustrated in Figure A1. The form of the fluxes are:

(A2) $$\begin{array}{l} F_{i-1/2,j}^{\left(n\right)}=\theta_{i-1/2,j}^{\left(n\right)}u_{i-1/2,j}^{\left(n\right)}\Delta t\Delta y\\ F_{i+1/2,j}^{\left(n\right)}=\theta_{i+1/2,j}^{\left(n\right)}u_{i+1/2,j}^{\left(n\right)}\Delta t\Delta y\\ F_{i,j-1/2}^{\left(n\right)}=\theta_{i,j-1/2}^{\left(n\right)}v_{i,j-1/2}^{\left(n\right)}\Delta t\Delta x\\ F_{i,j+1/2}^{\left(n\right)}=\theta_{i,j+1/2}^{\left(n\right)}v_{i,j+1/2}^{\left(n\right)}\Delta t\Delta x \end{array}$$

Equation (A2) lets us know that the fluxes are actually the gas volume flowing out of the cell. This gives the possibility to calculate the fluxes directly, instead of computing Equation (A2) from the interface geometry in the cell. There are several situations to discuss. If the computational method of any one of the fluxes is determined, it is easy to extend this to all others. Thus, we discuss $F_{i+1/2,j}^{\left(n\right)}$ as an example for the convenience of statements and understandings.

(1) If the cell is completely occupied by liquid ($\theta_{i,j}^{\left(n\right)}=0$), gas will not flow out of this cell thus that the flux is zero: $F_{i+1/2,j}^{\left(n\right)}=0$;

(2) If the cell is completely occupied by gas ($\theta_{i,j}^{\left(n\right)}=1$), gas flux can be easily calculated: $F_{i+1/2,j}^{\left(n\right)}=u_{i+1/2,j}^{\left(n\right)}\Delta t\Delta y$;

(3) If the cell contains both liquid and gas ($0<\theta_{i,j}^{\left(n\right)}<1$), the shadow triangle in Figure A2 represents the gas fraction $\theta_{i,j}^{\left(n\right)}$ while the white area represents liquid. The length *L*_1_ and the angle α of the triangle can be obtained in traditional VOSET method [38]. The shadow rectangular represents the fluid flows out from right the border. Two situations exist:

(3.1) $u_{i+1/2,j}^{\left(n\right)}\Delta t\leq \Delta x-L_{1}$ as shown in Figure A2a: All the fluid flowing out is liquid. That means no gas flows out from the right border, therefore $F_{i+1/2,j}^{\left(n\right)}=0$;

(3.2) $u_{i+1/2,j}^{\left(n\right)}\Delta t>\Delta x-L_{1}$ as shown in Figure A2b: The fluid flowing out consists of both liquid and gas. The gas volume flowing out from the right border can be represented by the area of the small triangle with the length *L*_2_. It is very easy to calculate *L*_2_ from the geometry: $L_{2}=u_{i+1/2,j}^{\left(n\right)}\Delta t-\left(\Delta x-L_{1}\right)$. Thus, $F_{i+1/2,j}^{\left(n\right)}=\frac{1}{2}L_{2}\left(L_{2}/\tan\alpha \right)$.

The above discussion is based on the assumption of $u_{i+1/2,j}^{\left(n\right)}>0$. If $u_{i+1/2,j}^{\left(n\right)}<0$, $F_{i+1/2,j}^{\left(n\right)}$ is not outer flux but inner flux for the cell (*i*,*j*) thus that the above method cannot be used. Fortunately, $F_{i+1/2,j}^{\left(n\right)}$ is the outer flux for the cell (*i*+1,*j*) as shown in Figure A2, thus that it can be computed using the above method in the cell (*i*+1,*j*). This means it is enough to only compute the outer fluxes for all the grid cells in the domain. Once the fluxes are computed, Equation (A1) can be used to update the gas fraction θ.
